# Trainees’ Exposure to the Field of Neurogastroenterology and Motility in Internal Medicine and General Surgery Residency Programs

**DOI:** 10.1111/nmo.70016

**Published:** 2025-03-27

**Authors:** Daniel L. Cohen, Amir Mari, Fahmi Shibli, Rita Brun, Tarek Arraf, Yoav Mazor, Nir Bar, Eran Ariam, Hagai Schweistein, Farouk Khatib, Vered Richter, Haim Shirin, Anton Bermont

**Affiliations:** ^1^ The Gonczarowski Family Institute of Gastroenterology and Liver Diseases Shamir Medical Center Zerifin Israel; ^2^ Faculty of Medical & Health Sciences Tel Aviv University Tel Aviv Israel; ^3^ Gastroenterology and Endoscopy Unit Nazareth Hospital EMMS Nazareth Israel; ^4^ Azrieli Faculty of Medicine Bar Ilan University Ramat Gan Israel; ^5^ Gastroenterology Department HaEmek Medical Center Afula Israel; ^6^ Rappaport Faculty of Medicine Technion Israel Institute of Technology Haifa Israel; ^7^ Gastroenterology Department Rambam Health Care Campus Haifa Israel; ^8^ Gastroenterology Department Tel Aviv Sourasky Medical Center Tel Aviv Israel; ^9^ Gastroenterology Department Kaplan Medical Center Rehovot Israel; ^10^ Division of Gastroenterology Rabin Medical Center Petah Tikva Israel; ^11^ Gastroenterology Department Bnai Zion Medical Center Haifa Israel

**Keywords:** gastrointestinal motility, gut–brain axis, medical education, neurogastroenterology, training programs

## Abstract

**Background:**

The disorders of neurogastroenterology and motility (NGM) are common, yet studies have shown that medical students have a relative lack of knowledge and confidence in this field, which may lead to poorer patient outcomes. We sought to evaluate whether this is also true of residents during the next stage of medical training.

**Methods:**

A questionnaire was developed and sent to internal medicine and general surgery trainees at nine teaching hospitals to assess their exposure to NGM and their comfort with the disorders of NGM versus organic gastrointestinal diseases.

**Results:**

A total of 121 trainees completed the questionnaire (mean age 32.7, 33.1% female, 71.9% internal medicine, and 28.1% general surgery). Overall, reported exposure to NGM was low (53.9%), mainly occurred during discussions on rounds, and was more common among surgeons (84.8% vs. 41.5%, *p* < 0.001). Overall, only 9.1% felt NGM was addressed at a moderate or high level, whereas only 13.3% felt knowledgeable enough to treat patients at a moderate or high level. Comfort with NGM diagnostic testing was also low, especially for anorectal manometry. When asked to rate their comfort with the pathophysiology, diagnosis, and treatment of eight diseases (4 NGM, 4 organic), comfort scores were significantly lower for the NGM disorders across all three domains for the whole population, as well as for internal medicine and surgical trainees individually (all *p* < 0.003).

**Conclusions:**

Exposure to NGM during residency training is low, with trainees often feeling inadequately prepared. This appears to be worse for internal medicine trainees than for general surgery trainees. Strategies to increase exposure and knowledge of NGM during residency training are needed.

## Graphical abstract

Internal medicine and general surgery trainees reported low exposure to neurogastroenterology and motility (NGM) in their training programs. Additionally, their comfort levels with NGM disorders were significantly lower than with organic GI disorders.

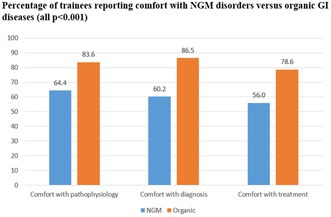

**Summary**

- Internal medicine and general surgery trainees report limited exposure to the field of neurogastroenterology and motility (NGM) during their training programs.
- They also report significantly less comfort with disorders of NGM in comparison to organic gastrointestinal disorders.

## 1 Introduction

Neurogastroenterology and motility (NGM) is the field within gastroenterology (GI) that deals with the interaction between the enteric nervous system and the digestive tract. The field of NGM includes disorders of gut–brain interaction (DGBI), formerly referred to as functional gastrointestinal disorders, which are extremely common. It has been shown that 40.3% of adults meet the criteria for at least one DGBI [1], and 34.9% of new referrals to a GI clinic are diagnosed with a DGBI [2]. Further, DGBI constitute a significant economic burden on the health care system [3], and patients with DGBI suffer from impaired quality of life [4].

Unfortunately, clinicians often have a less favorable perception of DGBI compared with organic GI diseases [5, 6]. This may be related to the lack of disease biomarkers leading to diagnostic uncertainty, a paucity of effective treatments, and the relative lack of personal and research renumeration [7, 8]. This is important as the negative clinician‐held perceptions may lead to worse healthcare outcomes, decreased patient satisfaction, and unnecessary healthcare utilization [9, 10, 11, 12].

The hope is that with better teaching and training in the realm of NGM, new physicians will better be able to address these issues and improve patient care [13]. However, several studies have shown that there is a relative lack of training, knowledge, and confidence in the realm of NGM among medical students [7, 14, 15]. Not only is this an issue among students but these same issues with NGM and DGBI have also been noted among GI trainees themselves [16, 17, 18].

Although exposure to and confidence in NGM has been assessed in medical students and GI trainees, so far, no studies have evaluated NGM exposure among trainees in the intermediate stage between medical school and GI fellowship (internal medicine residency). Also, since general surgeons treat many disorders in the field of NGM, including achalasia, gastroesophageal reflux disease (GERD), gastroparesis, and defecatory disorders, it is surprising that no studies have evaluated the exposure of general surgery residents to NGM.

We therefore created a questionnaire to assess internal medicine and general surgery trainees’ exposure to NGM, their comfort with aspects of DGBI, and their experiences with GI motility diagnostic testing. Our primary aim was to compare the comfort these trainees have with disorders of NGM versus organic GI diseases. Secondarily, we sought to assess whether and how they were exposed to NGM during their training. Finally, we aimed to compare the results between internal medicine and general surgery trainees.

## 2 Materials and Methods

### 2.1 Study Design and Ethical Considerations

This was a prospective, multicenter questionnaire study involving nine teaching hospitals located throughout Israel, spanning both rural and urban centers, and serving diverse populations. The participating medical centers included Shamir Medical Center, Nazareth Hospital EMMS, HaEmek Medical Center, Rambam Health Care Center, Tel Aviv Sourasky Medical Center, Rabin Medical Center, Meir Medical Center, Bnai Zion Medical Center, and Kaplan Medical Center.

The study was approved by the local Institutional Review Board (approval # 0019‐24‐ASF on 7 April 2024) and conducted according to the Declaration of Helsinki. No informed consent was required given the questionnaire's anonymous nature and that completing the questionnaire implied consent.

### 2.2 Questionnaire

An anonymous questionnaire was developed with input from leaders in the field of NGM from the participating medical centers. The goal of the questionnaire was to assess internal medicine and general surgery trainees’ exposure to and comfort with disorders of NGM (see Supporting Information 1).

The questionnaire contained several sections. The first section requested demographic data from the participant, including age, gender, residency field (internal medicine or general surgery), and year of training. The second section addressed the trainees’ exposure to NGM. The third section assessed their subjective comfort with the pathophysiology, diagnosis, and treatment of NGM disorders and organic GI diseases. Four disorders were chosen to represent each type based on a previous study [14]. For disorders of NGM, achalasia, GERD, gastroparesis, and irritable bowel syndrome (IBS) were chosen, whereas peptic ulcer disease (PUD), celiac disease, ulcerative colitis, and colorectal cancer were chosen to represent organic GI disorders. The final section of the questionnaire assessed the trainees’ exposure to several diagnostic tests within the field of NGM (esophageal manometry, pH‐impedance testing, gastric emptying scan, and anorectal manometry).

The questionnaire was revised several times by the authors until a final version was agreed upon. The final questionnaire was made available via Google Sheets. It was estimated that the questionnaire would take 7–10 min to complete. No single question was required to be answered to submit the questionnaire.

### 2.3 Trainee Participants

In Israel, internal medicine training is a 4‐year program consisting entirely of inpatient rounds without outpatient clinics. Trainees do have elective time, but only a small fraction chooses to participate in a GI elective. General surgery training is a 6‐year program and does include exposure to outpatient care. In the study, trainees were considered to be in the first half of their training program if they were in the first 2 years of internal medicine residency or the first 3 years of general surgery residency.

A link to the online questionnaire was sent to the e‐mail or WhatsApp list of internal medicine and general surgery trainees at each participating hospital. After a week, the link to the questionnaire was re‐sent a second time, and finally, a third request was sent after another week. Trainees were informed that the questionnaire was voluntary. The questionnaire was completed by trainees at the various hospitals between May and September 2024. Trainees in internal medicine and general surgery training programs were encouraged to complete the questionnaire. However, trainees in advanced subspecialty training programs beyond regular internal medicine and general surgery training, such as endocrinology, cardiology, or thoracic surgery fellowships, were not included.

### 2.4 Outcomes

The primary outcome was a comparison of the four chosen NGM disorders (achalasia, GERD, gastroparesis, IBS) versus the four organic GI disorders (PUD, celiac disease, ulcerative colitis, colorectal cancer) in terms of the comfort trainees expressed with the pathophysiology, diagnosis, and treatment of these disorders. The level of comfort expressed by the trainees was purely subjective. No attempt was made to objectively assess the trainees’ knowledge in these fields. Other outcomes assessed how trainees were exposed to NGM during their training, including their exposure to diagnostic testing. Finally, comparisons were made between the internal medicine trainees and general surgery trainees.

### 2.5 Statistical Analyses

Trainees reported their comfort with various diseases via four answer options (“uncomfortable,” “somewhat uncomfortable,” “somewhat comfortable,” “comfortable”). For statistical purposes, the two “uncomfortable” answers were grouped together, as were the two “comfortable” responses. Additionally, for comparisons between organic and NGM disorders, the responses for all four disorders within each grouping were grouped together. The four NGM disorders were achalasia, GERD, gastroparesis, and IBS, whereas the four organic GI disorders were PUD, celiac disease, ulcerative colitis, and colorectal cancer.

Continuous variables were reported as means with standard deviations when normally distributed; otherwise, they were reported as medians with interquartile ranges. For comparing categorical variables, Pearson χ^2^ test was used. However, for cases in which the number of variables was low, the Fisher exact test was used. For comparisons within groups, the paired t‐test was used. For continuous variables, the Mann–Whitney *U* test was performed. Statistical analysis was performed using SPSS version 26 for Windows (IBM SPSS Statistics, Armonk, NY). All statistical tests were 2‐sided, with *p* < 0.05 considered significant.

## 3 Results

### 3.1 Response Rate and Demographics of the Study Population

At the time of the study, there were a total of 290 internal medicine and 98 general surgery trainees at the nine participating hospitals. A total of 121 trainees completed the questionnaire, including 87 (71.9%) internal medicine and 34 (28.1%) general surgery trainees. Thus, the response rate was 30.0% for internal medicine, 35.7% for general surgery, and 31.2% overall.

The demographics of the study participants is presented in Table 1. The mean age was 32.7 ± 3.4 years old, and 40 (33.1%) were female. Fifty‐six (46.7%) were in the first half of their training program. No significant differences were noted between internal medicine and general surgery trainees in terms of the demographic data.

**TABLE 1:** Demographics of the study population.

	Overall	Internal Medicine	General Surgery	*p*‐value
	(*n* = 121)	(*n* = 87)	(*n* = 34)	
Age – years ± SD	32.7 +/− 3.4	32.4 ± 3.2	33.7 ± 3.8	0.085
Female – *n* (%)	40 (33.1%)	30 (34.5%)	10 (29.4%)	0.594
Trainees in the first half of their training program	56 (46.7%)	38 (44.2%)	18 (52.9%)	0.386

### 3.2 Exposure to Neurogastroenterology

**FIGURE 1:**
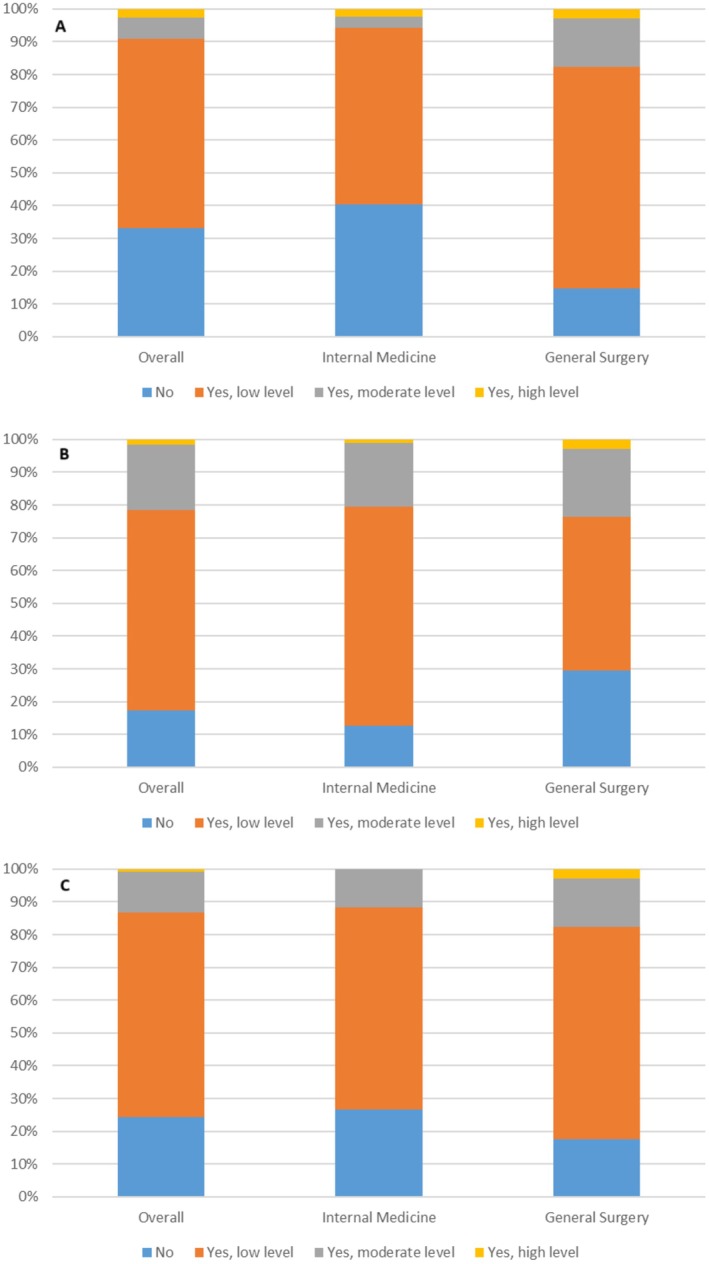
Exposure and comfort with NGM. (A) Has NGM been adequately addressed in your training program? (B) Was NGM adequately addressed in your medical school? (C) Do you feel comfortable enough to treat patients with NGM disorders?.

Overall, only 62 (53.9%) trainees reported exposure to NGM during their training program so far (Table 2). This was mainly in the form of discussions while on rounds (55, 45.5%), and less commonly due to lectures (15, 12.4%) or journal club (15, 12.4%). When asked if they felt that NGM had been adequately addressed in their training program (Figure 1A), the vast majority answered either “No” (40, 33.1%) or “Yes, but at a low level” (70, 57.9%). Only a total of 11 (9.1%) felt that NGM had been addressed at a moderate or high level. In comparison, these numbers were worse than when the trainees were asked about their experience while in medical school (Figure 1B). There, fewer respondents reported that NGM was not adequately addressed (21, 17.4%) and more reported that it was addressed at a moderate or high level (26, 21.5%).

**TABLE 2:** Trainees reporting exposure to neurogastroenterology, overall and by type.

	Overall	Internal medicine	General surgery	*p*‐value
Exposure to NGM in training	62 (53.9%)	34 (41.5%)	28 (84.8%)	< 0.001
**Type of exposure**				
Lecture attendance	15 (12.4%)	7 (8.0%)	8 (23.5%)	0.030
Journal club attendance	15 (12.4%)	5 (5.7%)	10 (29.4%)	< 0.001
Discussion on rounds	55 (45.5%)	29 (33.3%)	26 (76.5%)	< 0.001
Other type	25 (20.7%)	18 (20.7%)	7 (20.6%)	0.990

When asked if they feel knowledgeable enough to treat patients with disorders of NGM (Figure 1C), 29 (24.2%) said “No” with the majority (75, 62.5%) responding “Yes, but at a low level.” Only 16 (13.3%) felt knowledgeable enough at a moderate or high level.

Interestingly, there were clear differences between internal medicine and general surgery trainees regarding some of these questions. General surgery trainees reported higher levels of exposure to NGM overall compared with internal medicine trainees (84.8% vs. 41.5%, *p* < 0.01), including more exposure via lectures (23.5% vs. 8.0%, *p* = 0.030), journal club (29.4% vs. 5.7%, *p* < 0.001), and discussions on rounds (76.5% vs. 33.3%, *p* < 0.001). They also reported feeling that NGM was more adequately addressed in their training program (*p* = 0.016).

### 3.3 Exposure to NGM Diagnostic Testing

Trainees were asked to rate their exposure to four different diagnostic tests used in the field of NGM—esophageal manometry, pH‐impedance testing, gastric emptying scans, and anorectal manometry (Table 3). The majority of trainees had some exposure to esophageal manometry (76, 62.8%), but less than half had exposure to pH‐impedance testing or gastric emptying scans (both 54, 44.6%), and only a small number had exposure to anorectal manometry (33, 27.3%). For all four tests, most of the exposure to these diagnostic tests was via reading procedure reports, while actually witnessing the test or watching a video about the test was much less common. When asked about their comfort with the indications for these tests, less than half reported comfort for each of the four tests, with anorectal manometry receiving the lowest comfort rates (Table 3). Similarly, when asked about their comfort with interpreting these tests, comfort rates were even lower than for the indications, with anorectal manometry again receiving the lowest score.

**TABLE 3:** Exposure to neurogastroenterology diagnostic testing.

	Overall	Internal medicine	General surgery	*p*‐value
**Exposure to esophageal manometry**	76 (62.8%)	49 (56.3%)	27 (79.4%)	0.018
Read a test report	62 (51.2%)	38 (43.7%)	24 (70.6%)	0.008
Watched a video	25 (20.7%)	14 (16.1%)	11 (32.4%)	0.047
Witnessed the test	8 (6.6%)	6 (6.9%)	2 (5.9%)	> 0.999
**Exposure to pH‐impedance**	54 (44.6%)	33 (37.9%)	21 (61.8%)	0.018
Read a test report	43 (35.5%)	25 (28.7%)	18 (52.9%)	0.012
Watched a video	16 (13.2%)	8 (9.2%)	8 (23.5%)	0.069
Witnessed the test	4 (3.3%)	2 (2.3%)	2 (5.9%)	0.314
**Exposure to gastric emptying scan**	54 (44.6%)	32 (36.8%)	22 (64.7%)	0.005
Read a test report	43 (35.5%)	29 (33.3%)	14 (41.2%)	0.418
Watched a video	14 (11.6%)	4 (4.6%)	10 (29.4%)	< 0.001
Witnessed the test	5 (4.1%)	2 (2.3%)	3 (8.8%)	0.134
**Exposure to anorectal manometry**	33 (27.3%)	15 (17.2%)	18 (52.9%)	< 0.001
Read a test report	26 (21.5%)	11 (12.6%)	15 (44.1%)	< 0.001
Watched a video	7 (5.8%)	3 (3.4%)	4 (11.8%)	0.096
Witnessed the test	4 (3.3%)	1 (1.1%)	3 (8.8%)	0.067
**Comfort with the indications for:**				
Esophageal manometry	42 (34.7%)	26 (29.9%)	16 (47.1%)	0.074
pH‐impedance	44 (36.7%)	30 (34.9%)	14 (41.2%)	0.519
Gastric emptying scan	47 (39.2%)	33 (38.4%)	14 (41.2%)	0.777
Anorectal manometry	33 (27.5%)	16 (18.6%)	17 (50.0%)	0.001
**Comfort with interpreting:**				
Esophageal manometry	37 (30.8%)	24 (27.9%)	13 (38.2%)	0.270
pH‐impedance	31 (26.3%)	19 (22.6%)	12 (35.3%)	0.157
Gastric emptying scan	32 (27.1%)	17 (20.2%)	15 (44.1%)	0.008
Anorectal manometry	18 (15.1%)	8 (9.4%)	10 (29.4%)	0.006

Finally, when compared with internal medicine trainees, general surgery trainees reported significantly more exposure to all four of these diagnostic tests (all *p* < 0.05). Additionally, general surgery trainees reported higher rates of comfort with the indications for anorectal manometry (50.0% vs. 18.6%, *p* = 0.001) and comfort with the interpretation of gastric emptying scans (44.1% vs. 20.2%, *p* = 0.008) and anorectal manometry (29.4% vs. 9.4%, *p* = 0.006).

### 3.4 Comfort With Specific NGM and Organic GI Disorders

Table 4 shows the percentage of trainees reporting feeling comfortable with various GI diagnoses. In terms of comfort with the pathophysiology of disease, the highest comfort level went to PUD (90.1%) with the lowest score for IBS (43.8%). The same was true in terms of the trainees’ comfort with diagnosing the disorders, of which PUD again had the highest comfort rate (92.4%), whereas gastroparesis (48.3%) and IBS (49.6%) had the lowest rates. Finally, in terms of comfort with the treatment of the disorders, similar responses were reported to the other categories. PUD had the highest comfort rate (92.4%), whereas IBS (38.7%) had the lowest. Overall, comfort rates were higher for the organic GI disorders in comparison with the NGM disorders.

**TABLE 4:** Trainees reporting comfort with the pathophysiology, diagnosis, and treatment of specific NGM and organic GI diseases.

	Overall	Internal medicine	General surgery	*p*‐value
**Comfort with pathophysiology**				
NGM Disorders				
Achalasia	87 (71.9%)	61 (70.1%)	26 (76.5%)	0.484
Gastroesophageal reflux disease	100 (84.0%)	71 (82.6%)	29 (87.9%)	0.478
Gastroparesis	70 (58.3%)	46 (53.5%)	24 (70.6%)	0.087
Irritable bowel syndrome	53 (43.8%)	37 (42.5%)	16 (47.1%)	0.652
Organic GI Disorders				
Peptic ulcer disease	109 (90.1%)	77 (88.5%)	32 (94.1%)	0.506
Celiac disease	97 (80.2%)	74 (85.1%)	23 (67.6%)	0.031
Ulcerative colitis	103 (85.8%)	73 (84.9%)	30 (88.2%)	0.776
Colorectal cancer	95 (78.5%)	64 (73.6%)	31 (91.2%)	0.034
**Comfort with diagnosis**				
NGM Disorders				
Achalasia	76 (63.3%)	54 (62.8%)	22 (64.7%)	0.844
Gastroesophageal reflux disease	95 (79.2%)	68 (79.1%)	27 (79.4%)	0.967
Gastroparesis	57 (48.3%)	35 (41.7%)	22 (64.7%)	0.023
Irritable bowel syndrome	59 (49.6%)	43 (50.6%)	16 (47.1%)	0.728
Organic GI Disorders				
Peptic ulcer disease	110 (92.4%)	77 (90.6%)	33 (97.1%)	0.286
Celiac disease	89 (75.4%)	70 (83.3%)	19 (55.9%)	0.002
Ulcerative colitis	107 (89.9%)	74 (87.1%)	33 (97.1%)	0.175
Colorectal cancer	104 (88.1%)	70 (83.3%)	34 (100%)	0.010
**Comfort with treatment**				
NGM Disorders				
Achalasia	70 (59.3%)	45 (53.6%)	25 (73.5%)	0.046
Gastroesophageal reflux disease	102 (86.4%)	70 (83.3%)	32 (94.1%)	0.148
Gastroparesis	47 (39.8%)	27 (32.1%)	20 (58.8%)	0.007
Irritable bowel syndrome	46 (38.7%)	34 (40.0%)	12 (35.3%)	0.634
Organic GI Disorders				
Peptic ulcer disease	109 (92.4%)	75 (89.3%)	34 (100%)	0.058
Celiac disease	88 (75.2%)	71 (84.5%)	17 (51.5%)	< 0.001
Ulcerative colitis	84 (71.8%)	56 (67.5%)	28 (82.4%)	0.119
Colorectal cancer	89 (74.8%)	57 (67.1%)	32 (94.1%)	0.002

Next, comparisons were performed between the internal medicine and general surgery trainees. While internal medicine residents had higher comfort rates for celiac disease than general surgery trainees in all three categories, the general surgery trainees reported higher scores for multiple other diseases (see Table 4). Surgeons had higher comfort rates with the pathophysiology of colorectal cancer (CRC), the diagnosis of both gastroparesis and CRC, and the treatment of both gastroparesis and CRC.

### 3.5 Comfort With NGM Disorders Versus Organic GI Disorders

**FIGURE 2:**
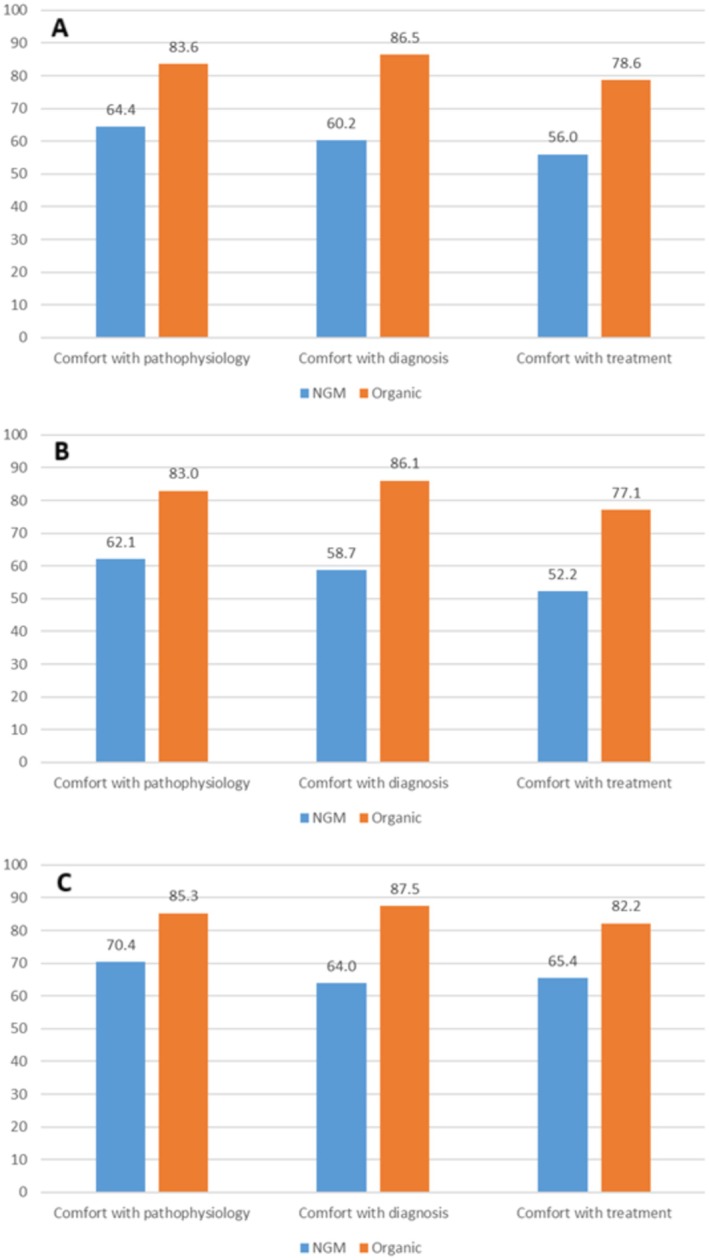
Percentage of trainees reporting comfort with NGM disorders versus organic GI diseases (composite of four NGM disorders and four organic GI disorders). (A) All participants. (B) Internal Medicine trainees. (C) General Surgery trainees.

We calculated an overall percentage of responses reporting comfort with the NGM disorders and organic GI disorders based on the four individual disorders within each category (Table 5 and Figure 2). For the whole population, rates of comfort were significantly higher for organic GI disorders than for NGM disorders across all three domains (pathophysiology: 83.6% vs. 64.4%, *p* < 0.001; diagnosis: 86.5% vs. 60.2%, *p* < 0.001; treatment: 78.6% vs. 56.0%, *p* < 0.001).

**TABLE 5:** Trainees reporting comfort with NGM disorders versus organic GI disorders (composite of four NGM disorders versus four organic GI disorders).

	Overall	Internal medicine	General surgery	*p*‐value medicine vs. surgery
**Comfort with pathophysiology**				
NGM disorders	310 (64.4%)	215 (62.1%)	95 (70.4%)	0.090
Organic GI disorders	404 (83.6%)	288 (83.0%)	116 (85.3%)	0.539
*p*‐value NGM vs. Organic GI	< 0.001	< 0.001	0.002	
**Comfort with diagnosis**				
NGM disorders	287 (60.2%)	200 (58.7%)	87 (64.0%)	0.284
Organic GI disorders	410 (86.5%)	291 (86.1%)	119 (87.5%)	0.686
*p*‐value NGM vs. Organic GI	< 0.001	< 0.001	< 0.001	
**Comfort with treatment**				
NGM disorders	265 (56.0%)	176 (52.2%)	89 (65.4%)	0.009
Organic GI disorders	370 (78.6%)	259 (77.1%)	111 (82.2%)	0.219
*p*‐value NGM vs. Organic GI	< 0.001	< 0.001	0.002	

Next, we evaluated if these higher rates of comfort with organic GI diseases compared with NGM disorders were dependent on the field of training. For all three domains (pathophysiology, diagnosis, and treatment), both internal medicine and general surgery trainees independently reported significantly higher comfort rates for organic diseases versus NGM disorders (all *p* < 0.003).

Finally, we compared comfort rates between internal medicine and surgery trainees. In all categories, general surgery trainees reported higher rates of comfort, although this was only significant for comfort with the treatment of NGM disorders (65.4% vs. 52.2%, *p* = 0.009).

## 4 Discussion

This study is the first to attempt to evaluate the exposure and comfort of internal medicine and general surgery trainees with the field of NGM. We found that trainees had little exposure to NGM, including NGM diagnostic testing, and consistently reported less comfort with disorders of NGM when compared with organic GI diseases.

The fact that trainees at this stage of their medical training reported poor exposure to NGM is not surprising, because this has been demonstrated at all stages of medical training that have been assessed [7, 13, 14, 15, 16, 17, 18]. A recent study from Spain showed that many gastroenterologists and GI fellows reported low satisfaction with their training in NGM during their GI training program and that the level of satisfaction was directly related to the number of weeks spent learning NGM during their training [18]. In regards to medical students, Patejdl et al. evaluated the representation of NGM within the curriculum of different medical schools in Germany [14]. They found that only 38% of students remembered NGM being covered in their curriculum, with similar findings at each medical school. Along with this, they found that students had the lowest competency ratings for IBS compared with other disorders, but those students who remembered being taught NGM had higher competency ratings. These findings highlight the connection between a lack of teaching NGM and poorer competency in treating patients with these disorders. Henick et al. in a study of medical students in New York, showed that some of the biases against NGM and DGBI begin as early as medical school in which students already have more negative attitudes towards patients with IBS when compared with inflammatory bowel disease [15]. They also showed that the more familiar one is with IBS, the less negative attitudes there are.

Although these studies show that additional exposure to NGM could increase young physicians’ comfort and confidence in treating patients with DGBI, this does not appear to be happening during post‐medical school training. We found that exposure to NGM was low at only 53.9% overall. Part of this may be explained by the fact that these trainees are still in the middle of their training program and still have time to learn about this field. However, this appears unlikely to explain most of the problem. When trainees did report exposure to NGM, most of it was in the form of discussions on rounds about specific patients. This implies that it was not necessarily a structured part of their training curriculum but rather randomly occurred based on which patients were admitted to their ward. If NGM is not enshrined in the curriculum of the training programs, many trainees will not be exposed to it, and if they are, it may be at a lower level. Also, as training programs are hospital‐based, there is often less exposure to ambulatory disorders, and DGBI tend to be more often treated on an outpatient rather than inpatient basis [1, 2, 3, 4]. This again may explain the low exposure in training programs. In fact, the trainees themselves rated their exposure to NGM as even less than in their medical school experiences. If the hope was that after medical school training programs would teach early‐stage physicians about NGM—conditions that affect up to 40% of the population [1]—this does not appear to be occurring.

As has been previously shown in other stages of medical training, low exposure to NGM leads to less comfort and confidence in treating these disorders. Our results show the same for both internal medicine and general surgery trainees as both groups reported lower comfort in the pathophysiology, diagnosis, and treatment of the NGM disorders than the organic diseases. While this was not objectively measured, the universally reported lack of comfort with these topics should be taken seriously. Specifically, comfort rates were consistently the lowest for IBS, an extremely common disorder for which non‐GI specialists should be expected to be knowledgeable. This finding is especially concerning.

One unique feature of our study was its assessment of NGM diagnostic testing, something which has not been previously reported. As opposed to the lecture halls of medical school, trainees are in hospitals and theoretically can witness and participate in diagnostic testing, which may provide them with exposure to new fields. However, our results show that few trainees had participatory exposure to these tests, with most exposure coming mainly from reading test reports. Interestingly, exposure was highest to esophageal manometry and lowest to anorectal manometry. Part of this may be the effects of the Chicago classification, which has created a uniform and systematic way to conduct and interpret esophageal manometry, possibly making it more interesting and understandable to trainees [19]. On the other hand, significantly fewer trainees were exposed to anorectal manometry, which also had the lowest scores for comfort in terms of indications and interpretation. This may be because anorectal manometry has traditionally tended to be less systematic and uniform in the way it is conducted and interpreted, although the London classification is attempting to address this [20].

Finally, we noted that these issues with NGM all tended to be worse for internal medicine trainees when compared with general surgery trainees. At first, this was surprising to us. We figured that since those interested in becoming gastroenterologists would go through the internal medicine pathway, at least some of those residents would have a strong basis in GI and might be more comfortable with NGM. However, studies have shown that even GI fellows have poor knowledge of NGM [16, 17]. Instead, it was the general surgery trainees who had more exposure to NGM and NGM diagnostic tests, as well as higher levels of comfort with NGM disorders. While we often think of NGM as a medical field, general surgeons have to be superbly knowledgeable about these disorders as well. General surgery has a large focus on the digestive tract, and patients with achalasia, GERD, gastroparesis, and defecatory disorders may all be treated surgically. It may be that the responsibility of operating on a patient compels surgeons, and surgical trainees as well, to understand the disease process and diagnostic test results at a higher level—something that does not apply to internal medicine doctors. Also, surgeons may be more confident in general compared with other physicians. Lastly, surgical trainees may also have more exposure to outpatient care in clinics during training compared with internal medicine residents who tend to have very little outpatient exposure in Israel.

There are interventions that may provide solutions for the issues raised in this study. The addition of structured lectures on NGM topics to the training curriculum is a simple addition that may ensure that all trainees have at least a baseline exposure to NGM. Also, the addition of ambulatory clinic rotations may increase exposure to outpatients with NGM disorders. Increased exposure to NGM through these interventions may also increase recruitment to the field of GI and NGM specifically. Despite being positively perceived, a recent study showed that many internal medicine trainees do not end up going into GI, often due to lifestyle preferences [21]. As NGM tends to have a better lifestyle than other fields within GI that rely on emergent procedures, more exposure to NGM during internal medicine training may increase the number of trainees applying to GI fellowships with an interest in NGM as a future specialty.

Although this study benefitted from its multicenter design, there are some limitations. It was conducted in one country, so the results may not be generalizable to other locations. There may be issues with sampling bias, as the response rate was relatively low, and there was a higher percentage of men than expected. Moreover, there were a relatively modest number of general surgery trainees included, so comparisons to internal medicine trainees should be interpreted cautiously. The participants were asked about their comfort with disorders of NGM and in treating patients, but no objective measurements of their knowledge were assessed. Additionally, selection bias might occur since all participating hospitals have NGM services, while other small district hospitals might not have NGM units. However, we believe including residents from centers without NGM units would reaffirm our conclusions, as their trainees’ exposure may be even lower. Finally, while four NGM disorders and four organic GI diseases were selected, partially based on the disorders chosen in a previous study [14], these may not necessarily represent one's knowledge and comfort with an entire field.

## 5 Conclusions

Internal medicine and general surgery trainees reported low exposure to the field of NGM. Moreover, this low exposure translated to worse comfort scores for disorders of NGM when compared with organic GI diseases. General surgery trainees had more exposure and better comfort with NGM than internal medicine trainees. Strategies to increase exposure to NGM, such as the addition of structured lectures to the training curriculum and ambulatory clinic rotations, especially for internal medicine residents, are needed to increase their knowledge and comfort with NGM, hopefully leading to better patient outcomes.

## Author Contributions

**Daniel L. Cohen:** conception and design; data collection; analysis and interpretation; drafting the manuscript; critical revisions. **Amir Mari:** data collection; critical revisions. **Fahmi Shibli:** data collection; critical revisions. **Rita Brun:** data collection; critical revisions. **Tarek Arraf:** data collection. **Yoav Mazor:** data collection; critical revisions. **Nir Bar:** data collection; critical revisions. **Eran Ariam:** data collection. **Hagai Schweistein:** data collection; critical revisions. **Farouk Khatib:** data collection. **Vered Richter:** conception and design; critical revisions. **Haim Shirin:** conception and design; critical revisions. **Anton Bermont:** conception and design; analysis and interpretation; critical revisions.

## Financial Support

The authors have nothing to report.

## Conflicts of Interest

The authors declare no conflicts of interest.

## Supporting information


**Appendix S1:** Supporting Information.

## Data Availability

The data that support the findings of this study are available from the corresponding author upon reasonable request.
