# Supplementary material for: Trainees’ Exposure to the Field of Neurogastroenterology and Motility in Internal Medicine and General Surgery Residency Programs
Source: Neurogastroenterol Motil. 2025 Mar 27;37(6):e70016. doi: 10.1111/nmo.70016 (PMC12075901; doi:10.1111/nmo.70016)
Supplement: Supplementary file 1 — Appendix S1: Supporting Information. [file NMO-37-e70016-s001.docx]

**Supplemental file: The Questionnaire**

Section 1: Demographics

1. What is your age? _____
2. What is your gender?

Male Female

1. What training program are you in?

Internal Medicine General Surgery

1. What year are you in your training program?

1 2 3 4 5 6 7

1. What medical center do you work at?

Nazareth EMMS HaEmek Rambam Tel Aviv Bnai Zion Meir Rabin Kaplan Shamir

Section 2: Exposure to Neurogastroenterology

1. Has the subject of “Neurogastroenterology” or “Gastrointestinal Motility Disorders” been addressed in your residency training program?

Yes No Cannot remember

1. If so, in which way has “Neurogastroenterology” or “Gastrointestinal Motility Disorders” been addressed?

Lectures Discussions on rounds Journal club Other

1. Do you feel that “Neurogastroenterology” or “Gastrointestinal Motility Disorders” has been adequately addressed in your training program?

Yes, high level Yes, moderate level Yes, low level No

1. Do you feel that “Neurogastroenterology” or “Gastrointestinal Motility Disorders” was adequately addressed in your medical school curriculum?

Yes, high level Yes, moderate level Yes, low level No

1. Do you feel that you have enough knowledge to treat patients with gastrointestinal motility disorders?

Yes, high level Yes, moderate level Yes, low level No

Section 3: Comfort with Disorders of Neurogastroenterology and Motility

1. How comfortable do you feel regarding the pathophysiology of the following diseases?

4-Comfortable 3-Somewhat comfortable 2-Somewhat uncomfortable 1-Uncomfortable

- achalasia
- gastroesophageal reflux
- peptic ulcer disease
- gastroparesis
- celiac disease
- irritable bowel syndrome
- ulcerative colitis
- colorectal cancer

1. How comfortable do you feel regarding the diagnosis of the following diseases?

4-Comfortable 3-Somewhat comfortable 2-Somewhat uncomfortable 1-Uncomfortable

- achalasia
- gastroesophageal reflux
- peptic ulcer disease
- gastroparesis
- celiac disease
- irritable bowel syndrome
- ulcerative colitis
- colorectal cancer

1. How comfortable do you feel regarding the treatment of the following diseases?

4-Comfortable 3-Somewhat comfortable 2-Somewhat uncomfortable 1-Uncomfortable

- achalasia
- gastroesophageal reflux
- peptic ulcer disease
- gastroparesis
- celiac disease
- irritable bowel syndrome
- ulcerative colitis
- colorectal cancer

Section 4: Exposure to neurogastroenterology diagnostic testing

1. Have you had exposure to any of the following diagnostic tests during your residency training?

Witnessed it Saw a video Read a report No exposure

- esophageal manometry
- pH-impedance testing
- gastric emptying scan
- anorectal manometry

1. How comfortable do you feel regarding the indications for the following tests?

4-Comfortable 3-Somewhat comfortable 2-Somewhat uncomfortable 1-Uncomfortable

- esophageal manometry
- pH-impedance testing
- gastric emptying scan
- anorectal manometry

1. How comfortable do you feel interpreting the results of the following tests?

4-Comfortable 3-Somewhat comfortable 2-Somewhat uncomfortable 1-Uncomfortable

- esophageal manometry
- pH-impedance testing
- gastric emptying scan
- anorectal manometry
